# Fabrication of a Molecularly-Imprinted-Polymer-Based Graphene Oxide Nanocomposite for Electrochemical Sensing of New Psychoactive Substances

**DOI:** 10.3390/nano13040751

**Published:** 2023-02-16

**Authors:** Xue Jiang, Fangsheng Wu, Xiaoyu Huang, Shan He, Qiaoying Han, Zihua Zhang, Wenbin Liu

**Affiliations:** 1 Shanghai Key Laboratory of Crime Scene Evidence, Shanghai Research Institute of Criminal Science and Technology, Zhongshan North No. 1 Road, Shanghai 200083, China; 2 Key Laboratory of Synthetic and Self-Assembly Chemistry for Organic Functional Molecules, Shanghai Institute of Organic Chemistry, Chinese Academy of Sciences, 345 Lingling Road, Shanghai 200032, China

**Keywords:** molecularly imprinted polymers, graphene oxide, RAFT, new psychoactive substances, electrochemical sensor

## Abstract

As new psychoactive substances (commonly known as “the third generation drugs”) have characteristics such as short-term emergence, rapid updating, and great social harmfulness, there is a large gap in the development of their detection methods. Herein, graphite oxide (GO) was first prepared and immobilized with a reversible addition-fragmentation chain transfer (RAFT) agent, then a new psychoactive substance (4-MEC) was chosen as a template, and then the surface RAFT polymerization of methacrylamide (MAAM) was carried out by using azobisisobutyronitrile (AIBN) as an initiator and divinylbenzene (DVB) as a cross-linker. After the removal of the embedded template, graphene oxide modified by molecularly imprinted polymers (GO-MIPs) was finally obtained. Owing to the specific imprinted cavities for 4-MEC, the satisfactory selectivity and stability of the GO-MIP nanocomposite have been demonstrated. The GO-MIP nanocomposite was then used to fabricate the electrochemical sensor, which displayed a high selectivity in detecting 4-MEC over a linear concentration range between 5 and 60 μg mL^−1^ with a detection limit of 0.438 μg mL^−1^. As a result, the GO-MIPs sensor developed an accurate, efficient, convenient, and sensitive method for public security departments to detect illicit drugs and new psychoactive substances.

## 1. Introduction

Nowadays, there is a proliferation, at an unprecedented scale, of new illicit drug discoveries on a global scale. To get around existing drug laws, manufacturers are developing new analogs by the modification of the chemical structure of known compounds. As a result, new psychoactive substances (NPS) are on the increase, and the illicit drug market has continued to expand over the last decade [1,2,3]. Up until the end of 2021, a total of 1127 different NPS were ever reported by 134 countries worldwide to the United Nations Office on Drugs and Crime (UNODC), but only 302 of them are under international control so far [4]. The global popularity of NPS is rather ominous, and the associated serious public health hazard has been gaining more attention. With the development of chemical techniques, most NPS have a short currency as some of them might been replaced by other analogues with better effects in the market [4]. Analytical laboratories of public security departments are thus confronted by the challenge of having to detect the appearance of such NPS in time.

As one of the most popular NPS, synthetic cathinones are widely abused and often sold as “ecstasy” as they have central-nervous-system-stimulant and hallucinogenic effects [4,5]. Manufacturers of novel drugs keep looking for alternatives, and a cumulative number of 201 synthetic cathinones were reported to UNODC by 2021 [4]. UNODC has summarized and recommended different methods to identify and analyze synthetic cathinones in seized materials [6]. The conventional methods for synthetic cathinone detection are mainly mass spectrometry (MS) [7,8,9,10], nuclear magnetic resonance (NMR) spectroscopy [10,11,12], Fourier-transform infrared (FTIR) spectroscopy [12], and Raman spectroscopy [13]. However, most of the instruments mentioned above are too bulky to allow portability, and are not only expensive but also need time-consuming operation. Although several methods using handheld instruments have been developed for on-field drug analysis [14,15,16], research results in this area are limited and synthetic cathinone detection still requires sensitive and portable devices for rapid screening on-site.

With the advantages of high sensitivity, portability, the low cost of instrumentation, and simplicity for operators, the electrochemical approach is an in-field detection method that has prevailed in recent years [17]. To this end, the use of screen-printed electrodes were shown to detect synthetic cathinones and metabolites [18,19,20,21]. Previous reports [22] have shown that the affinity and selectivity of electrochemical sensors could be improved by molecularly imprinted polymers (MIPs) with the advantage of having synthetic polymeric recognition elements. Notably, it has been previously reported that MIPs can be developed as extraction and quantification material for NPS [23]. MIPs are polymeric synthetic receptors which can be tailored towards different templates, and this makes them a very powerful tool for the selective extract traces of targets from complex samples [24,25]. Therefore, the combination of electrochemical sensors and MIPs is an effective way to overcome the drawbacks of traditional methods for NPS detection, offering an excellent opportunity to develop rapid analytical approaches with high selectivity. 

Traditionally, MIPs were prepared by bulk polymerization, but the deep embedment of the recognition sites within the thick polymer network would inevitably result in high diffusion barriers, low-affinity binding, and low-rate mass transfer [26]. An efficient approach to overcome these limitations is to construct such molecular recognition sites on support materials, which is known as the surface-imprinting method [27]. Significantly, MIP film coated carbon nanomaterials have been developed in recent years as transducer platforms with good electrical conductivity [28]. Thanks to the characteristic high surface-to-volume ratio and durable mechanical strength of graphene and its derivatives, such as graphene oxide (GO), they have been extensively studied in terms of their applications in sensors [29,30]. It is noteworthy that Li et al. [31] reported the first MIP-functionalized GO hybrid material by reversible addition and fragmentation chain transfer (RAFT) polymerization. Compared with other living/control radical polymerization techniques, RAFT polymerization not only allowed the synthetic tailoring of polymers with narrow molecular weight distribution, controlled molecular weight, and diverse terminal functionality, but has also been performed under mild reaction conditions with a wide range of monomers. Therefore, the specially-designed polymer structure can been used to improve the features of molecular imprinting such as maximum adsorption capacity, selectivity, and sensitivity [32]. 

**Scheme 1:**
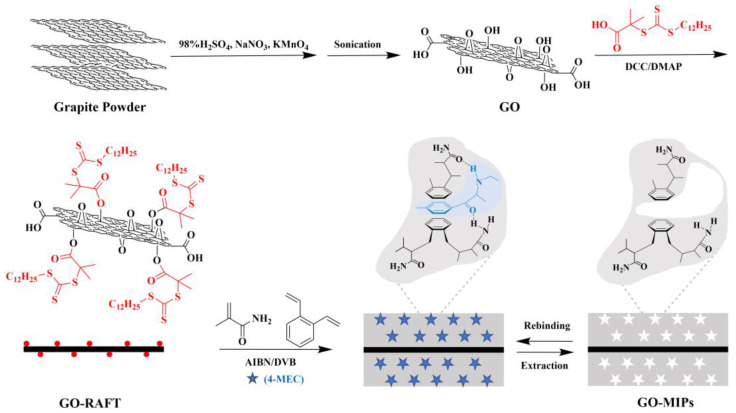
Schematic illustration of preparation of GO-MIP nanocomposites.

Herein, a sensitive electrochemical sensing platform based on GO was proposed for the detection of synthetic cathinones, in which 4-methylethcathinone (4-MEC) was used as the model abused drug. The GO-MIP nanocomposites were firstly fabricated by immobilizing MIPs onto the surface of GO through surface RAFT polymerization (Scheme 1): GO sheets were first prepared using a modified version of Hummer’s method [33,34], and then the RAFT agent was attached onto the surface of the GO through an esterification process. The MIP-modified GO nanocomposites were synthesized by a surface RAFT polymerization strategy using 4-MEC as a template, methacrylamide (MAAM) as a functional monomer, and divinylbenzene (DVB) as a cross-linker. The specific binding sites were achieved by multiple weak interactions (such as hydrogen bonds between amino-containing and amide-containing groups, and π-π bonds between phenyl groups). After removing the template, the selectivity and repeatability as well as the adsorption capacity of the obtained GO-MIP nanocomposites were investigated. In addition, an electrochemical sensor with a high adsorption capacity and excellent selective recognition ability was prepared. The results validated the feasibility of the obtained electrochemical sensor to detect 4-MEC with a low limit of detection (LOD).

## 2. Materials and Methods

### 2.1. Chemicals and Reagents

4-Ethylmethcathinone (4-EMC, 99%), mephedrone (4-MMC, 99%), 4-methyl-N,N-dimethylcathinone (4-MDMC, 99%), and tetrahydrofuranylfentanyl (THF-F, 99%) were supplied by Shanghai Yansi Standard Science and Technology Co., Ltd. (Shanghai, China). 2,2′-Azobis (isobutyronitrile) (AIBN, 98%, TCI, Shanghai, China) was recrystallized from anhydrous ethanol twice followed by drying at 25 °C in vacuum for one day. Graphite powder (99.99+%, Sigma-Aldrich, St. Louis, MO, USA), methacrylamide (MAAM, 98%, Sigma-Aldrich), divinylbenzene (DVB, 80%, Sigma-Aldrich), 2-(dodecylthiocarbonothioylthio)-2-methylpropionic acid (DDMAT, 98%, Sigma-Aldrich), dicyclohexylcarbodiimide (DCC, 99%, Adamas, Shanghai, China), 4-dimethylaminopyridine (DMAP, 99%, Adamas), methyl orange (MO, 98%, Adamas), permanganate (KMnO_4_, 99.5%, Hushi, Shanghai, China), sulfuric acid (H_2_SO_4_, 96%, Hushi), potassium hexacyanoferrate(III) (K_3_[Fe(CN)_6_], 99%, Adamas), and potassium chloride (KCl, 99.9%, Adamas) were used as received. Dichloromethane (DCM) and N,N-Dimethylformamide (DMF) were dried over CaH_2_ and distilled under reduced pressure before use. Tetrahydrofuran (THF) was distilled from sodium and benzophenone under nitrogen before use. Deionized water was obtained using a Milli-Q water purification system (Millipore, Burlington, MA, USA).

### 2.2. Measurements

Fourier-transform infrared (FT-IR) spectra were obtained by a PerkinElmer (Frontier) instrument (Waltham, MA, USA). Raman spectra were recorded on a HORIBA HR Evolution instrument (Tokyo, Japan). Scanning electron microscopy (SEM) images were recorded by a ZEISS GeminiSEM 300 instrument (Oberkochen, Germany). Transmission electron microscopy (TEM) images were recorded by a JEOL JEM 1230 instrument (Tokyo, Japan) operated at 80 kV. Cyclic voltammetry (CV) and differential pulse voltammetry (DPV) were performed on a Metrohm Autolab PGSTAT302N instrument (Herisau, Switzerland) and controlled by a microcomputer with Metrohm Autolab Nova software. High performance liquid chromatography (HPLC) was performed on a Shimadzu LC-20AT instrument (Kyoto, Japan).

### 2.3. Preparation of GO Sheets

Graphite powder(5.0 g) was firstly stirred in 98% sulfuric acid (230 mL) under an ice bath, and NaNO_3_ (5.0 g) and KMnO_4_ (5.0 g) were carefully added to the mixture to prevent the temperature from exceeding 15 °C. Then, the flask was placed in a water bath at about 35 °C for 30 min. Finally, 230 mL deionized water was slowly stirred into the mixture and maintained the temperature of the reaction solution within 100 °C. The mixture was centrifuged and washed repeatedly until it reached neutrality to remove residual acids and salts. The obtained GO dispersion (0.1 mg mL^−1^) was completely exfoliated by ultrasound for at least 3 h. The final GO sheets were recovered by filtration and vacuum drying.

### 2.4. Introduction of RAFT Functionalities onto GO Sheets

The GO sheets (1.0 g) were firstly dispersed in DMF (500 mL) for 15 min, followed by undergoing sonication for a further 15 min. Next, DDMAT (0.5 g) was introduced, and the solution was further stirred at room temperature for 5 min. After DCC (2.5 g) and DMAP (0.5 g) were added to the solution, the resultant mixture was continuously stirred at room temperature for 24 h. The solvent was then removed under reduced pressure, and the solid was washed with DCM four times to remove the unattached RAFT agent. Finally, the product (GO-RAFT) was dried in vacuo overnight.

### 2.5. Preparation of GO-MIPs

MAAM (1.02 g), DVB (8.2 mL), GO-RAFT (200 mg), and 4-MEC (200 mg) were dissolved in 20 mL THF, and the mixture was purged with nitrogen and then sealed. After adding 2.0 mg AIBN, the resultant reaction was performed at 55 °C for 24 h under a nitrogen atmosphere. To remove the template and unreacted reagents, the obtained GO-MIPs were washed three times with a solution of chloroform, methanol, and acetone. Finally, the resultant product (GO-MIPs) was dried in vacuo at 60 °C for 24 h. As a reference, the non-imprinted GO (GO-NIPs) was prepared by the same synthetic protocol in the polymerization process, only without adding 4-MEC.

### 2.6. Batch Mode Adsorption Studies

A certain amount (30 mg) of sorbent (GO-MIPs or GO-NIPs) was firstly added to 10 mL of a testing solution of 4-MEC (0.02 mmol L^−1^). Next, the testing solutions were treated in a batch mode of operations, and the concentration of 4-MEC in the solution was measured with HPLC. The equilibrium adsorption capacity (*Q_e_*, mg g^−1^) was then calculated according to the following equation:

(1) $$Q_{e}=\frac{\left(C_{0}-C_{e}\right)V}{W}$$

In Equation (1), *V* (mL) and *W* (g) are the volume of the testing solution and the weight of the sorbent, respectively. *C*_0_ (mg L^−1^) and *C_e_* (mg L^−1^) are the initial and equilibrium concentrations of 4-MEC in the solution, respectively. 

### 2.7. Fabrication Process of GO-MIPs Sensor

An amount of 3.0 mg GO-MIPs was first mixed with 1.0 mL solution (deionised water/ethanol = 3/7), and 25 μL Nafion was then added. An amount of 4.0 μL of GO-MIP solution prepared in advance was cast on a clean glassy carbon electrode (GCE) surface (d = 4 mm). The electrode modified by GO-MIP nanocomposites was subsequently used as the working electrode after water evaporation.

A non-imprinted sensor (GO-NIPs sensor), as a control, was prepared by using GO-NIPs instead of GO-MIPs with the same procedure. 

### 2.8. Electrochemical Experiments

All electrochemical experiments were carried out with a three-electrode system, which includes a reference electrode (saturated calomel electrode), a counter electrode (platinum wire electrode), and the working electrode (bare GCE or modified). Prior to each measurement, the bare glass carbon electrodes were polished with an alumina slurry (0.05 µm) and felt pad, followed by being thoroughly washed with acetone, HNO_3_, NaOH solution (1.0 mol L^−1^), and deionized water, and finally being dried under a nitrogen stream to obtain the mirror surface. The measurements of CV were recorded with the potential ranges of −0.2 to +0.8 V at a scan rate of 100 mV s^−1^ in a solution containing 5.0 mmol L^−1^ of K_3_[Fe(CN)_6_] and 0.1 mol of L^−1^ KCl. A scan potential from −0.2 to +0.6 V was used for DPV measurements. 

## 3. Results and Discussion

### 3.1. Characterization of the Obtained GO-MIP Nanocomposites

**Figure 1:**
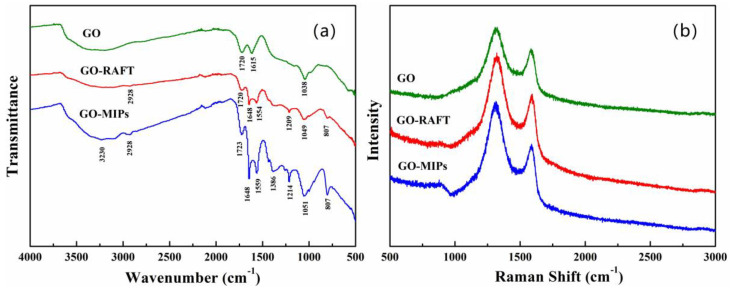
FTIR spectra (**a**) and Raman spectra (**b**) of GO, GO-RAFT, and GO-MIPs.

As FTIR spectroscopy is a powerful tool for identifying the chemical properties of carbon materials, the products of each reaction step were first characterized to ensure the successful preparation of GO-MIP nanocomposites. As shown in Figure 1a, the typical peaks of GO could be observed, such as the stretching vibrations of O-H, C=O, and C=C, centered at 3428, 1720, and 1615 cm^−1^, respectively. Compared with that of the GO, the characteristic absorbance band of GO-RAFT appeared at 2928 cm^−1^, which is related to the C-H stretching vibrations of the RAFT agent, while the intense bands at 1648, 1209, and 807 cm^−1^ are attributed to the stretching of C=O, C=S, and C-S, respectively. The peak at 3230 cm^−1^ in the GO-MIPs is ascribed to the -NH_2_ stretching vibration, which confirms that the grafted polymers were covalently bound to the surface of the GO. The structures of GO, GO-RAFT, and GO-MIPs were further investigated by Raman spectroscopy (Figure 1b), and two pronounced peaks were clearly visible at around 1325 cm^−1^ and 1583 cm^−1^, corresponding to the so-called D and G bands, respectively. The G band is characteristic of sp^2^-hybridized carbon networks, and the D band is related to the disorder caused by structural imperfections. Compared with that of GO, the integrated intensity ratio of the D and G bands (*I*_D_/*I*_G_) of GO-MIPs was higher, which suggests that the formation of molecularly imprinted polymers increased the disorder of the nanocomposites, and this is consistent with other studies [31].

**Figure 2:**
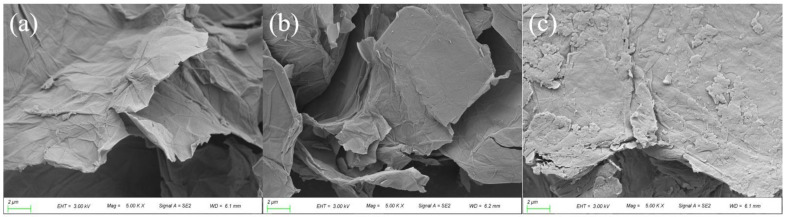
SEM images of the prepared GO sheets (**a**), GO-RAFT (**b**), and GO-MIPs (**c**).

**Figure 3:**
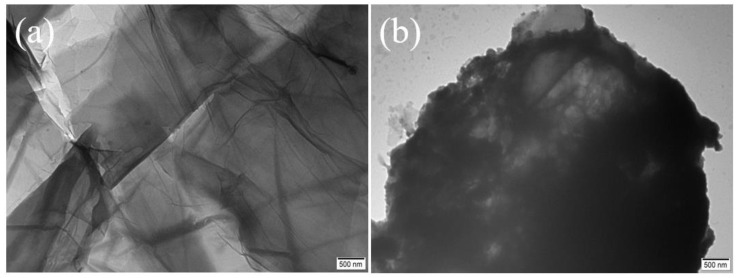
TEM images of the prepared GO sheets (**a**) and GO-MIPs (**b**).

The morphological structures of the prepared GO sheets and GO-MIPs were visually observed by SEM and TEM. Figure 2 shows SEM images of GO, GO-RAFT, and GO-MIPs, and the typical lamella structure of GO sheets with crumpled and wrinkled surfaces can be clearly observed in Figure 2a. The GO-RAFT looked like rough pieces of wrinkled flakes, as can be seen in Figure 2b, showing that the covalent connection of the small molecule RAFT reagent does not cause obvious changes in the surface morphology of GO sheets. Compared with the GO sheets and GO-RAFT, the GO-MIP nanocomposites had a dense and rough surface (Figure 2c), which indicated the successful formation of MIPs on the GO surface with a high polymerization efficiency. TEM has proven to be a very effective way to examine particle size and morphology before and after modification. From the TEM image (Figure 3a), it can be seen that the GO sheets exhibit an ultrathin structure and are like a very thin piece of paper with some wrinkles. As can be noticed in Figure 3b, the MIP films were widely and uniformly dispersed on GO surface, indicating that the imprinted cavities were formed on the basal planes of GO sheets [31].

### 3.2. Adsorption Behavior of GO-MIPs for 4-MEC

#### 3.2.1. Adsorption Kinetics

**Figure 4:**
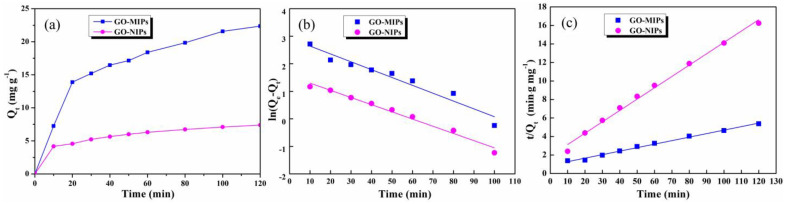
Dynamic adsorption (**a**), pseudo-first-order kinetic for adsorption (**b**), and pseudo-second-order kinetic for adsorption (**c**) of GO-MIPs and GO-NIPs.

In order to study the adsorption behavior of GO-MIPs towards the template (4-MEC), an adsorption kinetics analysis was first carried out. Figure 4a shows the dynamic adsorption curves of GO-MIPs, and the adsorption process proceeded in two steps. The existence of a large number of active binding sites on the external surface of GO-MIPs made the adsorption rate quite fast in the first step, and about 62% of the maximum adsorption amount of 4-MEC was taken up from the solution in 20 min. In the subsequent step, the adsorption was slow at reaching equilibrium, and the relatively long contact time could be associated with the fact that the 4-MEC in outer space had to diffuse to the inner cavities [35]. The maximum adsorption capacity of the experiment was gradually achieved within about 2 h, indicating the successful formation of the cavities with specific shapes in the GO-MIP nanocomposites during the imprinting process. For comparison, the adsorption capacity of the GO-NIPs was lower, which might only have been caused by non-specific electrostatic and hydrophobic interactions.

In order to examine the mechanism of the adsorption process, a pseudo-first-order rate equation and a pseudo-second-order rate equation have been used to analyze the kinetic data [36,37,38]. The results can be calculated according to the following equations:

(2) $$\ln\left(Q_{e}-Q_{t}\right)=\ln Q_{e}-k_{1}t$$

(3) $$\frac{t}{Q_{t}}=\frac{1}{Q_{e}^{2}k_{2}}+\frac{t}{Q_{e}}$$

In Equations (2) and (3), *t* is the adsorption time (min); *Q_e_* (mg g^−1^) is the capacity of the 4-MEC adsorbed at the equilibrium, and *Q_t_* (mg g^−1^) is the capacity of the 4-MEC adsorbed at the time, *t*. *k*_1_ and *k*_2_ mean the pseudo-first-order and pseudo-second-order rate constant, respectively. The plots of ln (*Q_e_* − *Q_t_*) vs. *t* (Figure 4b) gave a straight line, and the values of *k*_1_ and *Q_e_* could be obtained from the slope and intercept. Similarly, the slope and intercept of the plots of *t*/*Q_t_* vs. *t* (Figure 4c) were used to calculate the values of *k*_2_ and *Q_e_*. Table 1 shows the summarized adsorption rate constants and linear regression values. The linear correlation coefficient value (R^2^) of the pseudo-first order (0.9393) was obviously lower than the pseudo-second order (0.9927), indicating that the adsorption of 4-MEC onto GO-MIPs followed pseudo-second order kinetics. The results suggest that the adsorption process of GO-MIPs towards 4-MEC is a chemical process and that the adsorption rate is controlled by the movement of 4-MEC within the specific cavities of GO-MIPs [35]. Furthermore, it was noticed that the data of GO-NIPs also fitted well with the pseudo-second-order model, which may be attributed to the multi-interactions, such as the chemical and physical adsorption of 4-MEC and NIPs on the surface [36,39].

#### 3.2.2. Adsorption Isotherms

**Figure 5:**
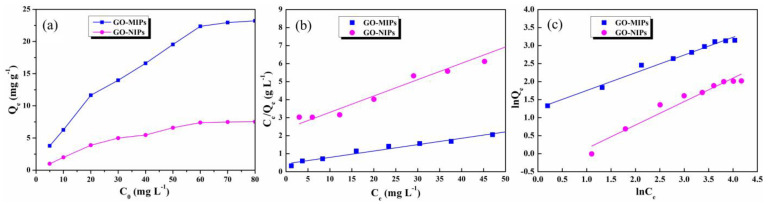
Adsorption isotherms (**a**), Langmuir (**b**), and Freundlich (**c**) isotherm models of GO-MIPs and GO-NIPs.

The 4-MEC recognition ability of GO-MIPs was further investigated by the static absorption experiments. As shown in Figure 5a, the equilibrium adsorption capacity (*Q_e_*) of 4-MEC increased quickly with the increase of the initial concentration at first, and then increased slowly until saturation. The maximum adsorption capacity of the 4-MEC onto the GO-MIPs was measured as 23.22 mg g^−1^, which was about 3 times higher than that of the control non-imprinted GO-NIPs (7.53 mg g^−1^). On the basis of the specific recognition of the imprinting cavities, GO-MIPs possessed a better adsorption ability to 4-MEC. The isotherm models of Langmuir and Freundlich were applied to describe the interaction between the target 4-MEC and GO-MIPs, and the applied linear model could be calculated using these equations [40]:

(4) $$\frac{C_{e}}{Q_{e}}=\frac{1}{Q_{m}K_{L}}+\frac{C_{e}}{Q_{m}}$$

(5) $$\ln Q_{e}=\frac{1}{n}\ln C_{e}+\ln K_{F}$$

In Equations (4) and (5), *C_e_* (mg L^−1^) is the equilibrium concentration of 4-MEC in solution, *Q_e_* (mg g^−1^) is the adsorption capacity of 4-MEC at equilibrium, *Q_m_* (mg g^−1^) is the maximum adsorption capacity, *K_L_* (L mg^−1^) is the Langmuir constant, and *K_F_* and *n* are the Freundlich constants. As described in Figure 5b, plotting *C_e_*/*Q_e_* against *C_e_* gives a straight line, and the values of *Q_m_* and *K_L_* can be obtained from the slope and intercept. In addition, the values of *K_F_* and *n* also can be obtained from the intercept and slope of the linearized plot of ln*Q_e_* vs. ln*C_e_* (Figure 5c). The calculated coefficients and constants were summarized in Table 2. As we know, the Langmuir isotherm model presumes an ideal adsorption process in which all the absorbing sites are homogenous and the adsorption is restricted only up to the mono-layer, while the Freundlich isotherm model describes the multi-layer adsorption of the adsorbate onto a heterogeneous surface [40]. The Freundlich model (R^2^ = 0.9842) is more adequate in describing the adsorption process than the Langmuir model (R^2^ = 0.9772) for 4-MEC adsorption onto GO-MIPs, suggesting that the adsorption in this study can be considered as multi-layer sorption. When the value of *n* is higher than 1, this indicates a favorable adsorption process [41]. In contrast, Langmuir isotherm (R^2^ = 0.9768) is concluded to be a better fit to describe the adsorption activity of GO-NIPs compared to Freundlich isotherm (R^2^ = 0.9489), which is probably because mono-layer adsorption mainly relies on the non-selective action of the surface and the fact that there is no selective binding point inside the GO-NIPs.

#### 3.2.3. Adsorption Selectivity

**Figure 6:**
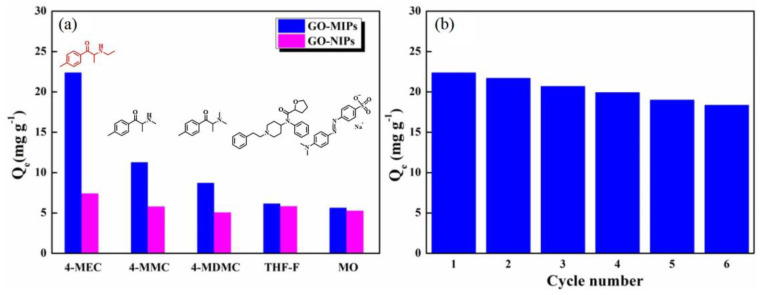
The selectivity of the GO-MIPs and GO-NIPs for adsorption of 4-MEC and interferents in aqueous medium (**a**), and repeatability experiments of GO-MIPs over six cycles of adsorption–desorption in methanol of the extraction at ambient temperature (**b**).

The experiment on the selective adsorption of the GO-MIPs towards 4-MEC was conducted by comparing it with the selective adsorption of GO-MIPs towards 4-MMC, 4-MDMC, THF-F, and MO. Among the four potential interferents, 4-MMC and 4-MDMC, which are both synthetic cathinones, are similar to 4-MEC in chemical structure to a certain extent. As shown in Figure 6a, the results prove that the selectivity performance of GO-MIPs towards 4-MEC is better than that of GO-MIPS towards the other four compounds, with a higher adsorption capacity. On the other hand, the adsorption capacity of GO-NIPs towards the five compounds is very small and shows no difference between the compounds, indicating that GO-NIPs have no selectivity. According to the above result, the template 4-MEC could been recognized by the specific cavities with molecular shape memory on the GO-MIPs, suggesting a high adsorption selectivity.

#### 3.2.4. Adsorption Repeatability

To evaluate the stability of the imprinted material, the adsorption repeatability experiment was carried out by using GO-MIPs six times. After every measurement, the GO-MIPs were washed with methanol to remove the 4-MEC molecules for reuse. Figure 6b displays that 82% of the initial value of the adsorption capacity was retained after six adsorption–desorption cycles, suggesting that the GO-MIP composites had acceptable stability under washing and in the incubation procedures, and that the subsequent GO-MIPs sensor could be reused at least six times for further selective recognition and separation. The slight decrease in adsorption capacity might have been because of the imprinted polymer defects, such as swelling and cracking, caused by the removal of the template several times. 

### 3.3. Electrochemical Behaviour of GO-MIPs

**Figure 7:**
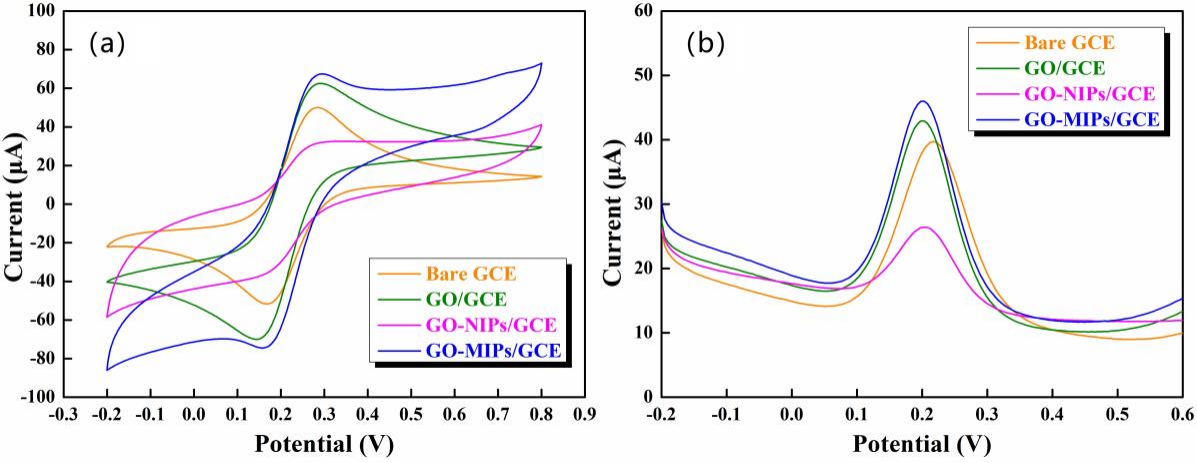
CV curves (**a**) and DPV curves (**b**) of bare GCE, GO/GCE, GO-NIPs/GCE, and GO-MIPs/GCE in a solution containing 5.0 mmol L^−1^ of K_3_[Fe(CN)_6_] and 0.1 mol L^−1^ KCl.

The modified electrodes were prepared by simply dropping the obtained GO-MIP nanocomposite dispersion on a freshly polished glass carbon electrode (GCE) and then drying it. The surface status and the barrier of the different modified electrodes could have been monitored by the CV using potassium ferrocyanide as a probe [42]. Figure 7a shows the CV responses of four different modified electrodes in a 5.0 mM K_3_[Fe(CN)_6_] solution and 0.1 M KCl. In the case of the bare GCE, the typical reversible process of the electrochemical response for [Fe(CN)_6_]^3−^ was observed. With the unique electric conductivity, the redox peak current was shown to increase for GO/GCE compared with bare GCE. As for GO-MIPs/GCE, the electrochemical behavior was equal to or slightly superior to GO/GCE, which could be explained by the possibility that the [Fe(CN)_6_]^3−^ molecules could easily reach the surface of the electrode through the vacant cavities. In contrast, the redox peak current of [Fe(CN)_6_]^3−^ declined dramatically after GCE was coated with GO-NIPs, which could be due to the weak conductivity of dense polymers. The successful fabrication of the GO-MIPs sensor was further verified by DPV, and the results were in good agreement with the CV results. As shown in Figure 7b, the distinct redox peak of [Fe(CN)_6_]^3−^ was found to be at around 0.2 V, and the peak current of GO-NIPs/GCE was significantly lower than those of bare GCE, GO/GCE, and GO-MIPs/GCE, providing direct evidence of vacant cavities in the GO-MIPs.

**Figure 8:**
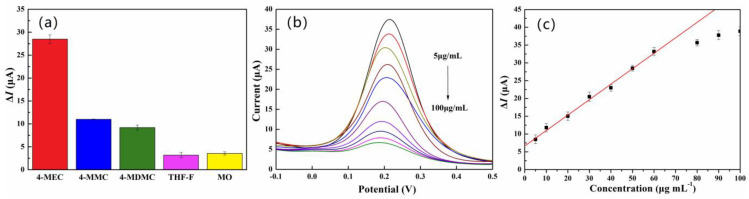
Selectivity of GO-MIPs/GCE toward different interferents (**a**), DPV responses of GO-MIPs/GCE for various concentrations of 4-MEC (**b**), and dependence of Δ*I* on the concentration of 4-MEC (**c**) in a solution containing 5.0 mmol L^−1^ of K_3_[Fe(CN)_6_] and 0.1 mol L^−1^ KCl.

As the performances of the GO-MIP composite modified electrode showed the advantages of good electrical conductivity, excellent selectivity, and high cycle stability, it possesses potential in the applications of electrochemical sensors. To further validate the selectivity of the GO-MIP composite modified electrochemical sensor, the current responses to 4-MEC, 4-MMC, 4-MDMC, THF-F, and MO were detected in a K_3_[Fe(CN)_6_] solution, respectively. When the vacant cavities were attached with the adsorbate molecules, the response current decreased, and as a result, the reduction peak current (Δ*I*) could be used to represent the adsorption ability of the imprint sites for different adsorbate molecules. As seen in Figure 8a, the electrochemical sensor showed a much larger value of Δ*I* for 4-MEC than for the other four potential interferents at the same concentration. DPV analysis was done for different concentrations of 4-MEC from 5 to 100 μg mL^−1^ under optimized conditions (Figure 8b). With an increase in the 4-MEC concentration, the redox peak current decreased significantly. The calibration curve presented in Figure 8c showed a linear relationship over the range of 5–60 μg mL^−1^ with the equation y = 0.4344x + 6.6320, and the correlation coefficient (R^2^) was demonstrated to be 0.9925. Moreover, the signal-to-noise (S/N) ratio was considered as 3 to estimate the limit of detection (LOD) for 4-MEC. The LOD was calculated as 0.438 μg mL^−1^ from the slope (m) and the standard deviation (S) was calculation by Equation (6).

(6) $$LOD=\frac{3S}{m}$$

## 4. Conclusions

In summary, we developed a facile method for preparing molecularly imprinted nanocomposites (GO-MIPs) by a surface RAFT polymerization strategy. The growth of uniform MIP film was validated via the FT-IR, Raman, SEM, and TEM characterization methods. The adsorption results indicated that the GO-MIPs possessed high selectivity towards 4-MEC, and that the presence of 4-MEC imprinted binding sites improved the adsorption performance. Kinetic and static adsorption tests concluded that the adsorption process of 4-MEC on GO-MIPs was best described using the pseudo-second-order and Freundlich model, indicating multi-layer molecule adsorption. Furthermore, the prepared GO-MIPs also possessed good selectivity and recyclability for 4-MEC molecules. Thanks to the large surface area and superior electrical conductivity of GO-MIPs, the GO-MIPs/GCE sensor exhibited a linear detection range (5–60 μg mL^−1^) and a LOD of 0.438 μg mL^−1^, making it attractive for the detection of NPS in the forensic science field.

## Figures and Tables

**Table 1:** Kinetic constants of the pseudo-first-order equation and pseudo-second-order equation for 4-MEC on GO-MIPs and GO-NIPs.

Samples	*Q_e_*_,exp_ (mg g^−1^)	Pseudo-First-Order			Pseudo-Second-Order		
		*k*_1_	*Q_e_* (mg g^−1^)	R^2^	*k*_2_	*Q_e_* (mg g^−1^)	R^2^
GO-MIPs	22.36	0.0285	18.690	0.9393	0.00159	26.455	0.9927
GO-NIPs	7.39	0.0261	4.759	0.9805	0.00789	8.157	0.9934

**Table 2:** Langmuir and Freundlich model adsorption constants of GO-MIPs and GO-NIPs for the adsorption of 4-MEC.

Samples	Langmuir			Freundlich		
	*Q_m_* (mg g^−1^)	*K_L_* (L mg^−1^)	R^2^	*K_F_*	*n*	R^2^
GO-MIPs	28.27	0.07989	0.9772	3.5323	2.0306	0.9842
GO-NIPs	11.02	0.03797	0.9768	0.6024	1.5356	0.9489

## Data Availability

The data presented in this study are available on request from the corresponding author.
